# Functionalization of Pasteurized Milk Using Rosemary, Thyme, and Ammoides Aqueous Extracts for Better Microbial Quality and an Improved Antioxidant Activity

**DOI:** 10.3390/molecules27123725

**Published:** 2022-06-09

**Authors:** Amel Ben Jalloul, Nourhene Ayadi, Amira Klai, Manef Abderrabba

**Affiliations:** 1 Laboratory of Materials Molecules and Applications (LMMA), Preparatory Institute of Scientific and Technical Studies (IPEST), University of Carthage, Tunis 2070, Tunisia; nourhenayadi90@gmail.com (N.A.); klaiamira00@gmail.com (A.K.); 2 National Agronomic Institute of Tunisia (INAT), University of Carthage, Tunis 2070, Tunisia

**Keywords:** medicinal plant, microbial quality, antioxidant activity, anti-listerial activity, milk

## Abstract

This study aimed to evaluate the effects of thyme (*Thymus vulgaris* L.), rosemary (*Rosmarinus officinalis* L.), and ammoides (*Ammoidespusilla* L.) aqueous extracts supplementation on the quality of heat-treated (55, 65, and 75 °C) raw milk (sample lots: A, B, and C) and cold-stored pasteurized milk (lot D). The three herbs have shown rich polyphenol contents (32.65–104.23 mgGAE/g), relevant antioxidant capacity, and high caffeic and rosmarinic acids and catechin contents. A significant reduction in psychrotrophic and total viable bacteria counts (PC and TVC) was attained following milk extract supplementation in both experiments. Monitoring PC and TVC counts in sample lots (A, B, and C) has revealed a significant improvement in extracts’ effectiveness in reducing the TVC count with the increment of temperature. The highest reductions of PC and TVC counts were achieved, respectively, in samples treated with thyme and rosemary in (lots: A, B, and C) and in samples treated with ammoides and thyme in (lot D). Thyme extract showed the highest milk serum DPPH scavenging activity (74.84% at 0.1 mg/mL) and induced a significant *Listeria monocytogenes* growth inhibition (>1 Log cycle). The sensory evaluation of supplemented milk has shown good consumer acceptability of ammoides-supplemented milk, quite similar to the control sample.

## 1. Introduction

Milk is one of the most extensively consumed food products thanks to its high nutritional value and relatively low price. In Tunisia, the dairy value chain is a strategic branch of the agricultural sector. The amount of collected and industrialized milk is 1.025500 tons in 2017 [1]. Raw milk, mainly sourced from small and primitive farms (80%), presents a poor microbial quality that does not meet the standard bacteriological quality criteria [1]. Thus, heat treatment must be applied in the early stage of the dairy-product processing chain. Indeed, pasteurization is a key step omnipresent in the production chains of the different dairy products. Pasteurization used for microbial decontamination of milk has been found to be effective in sustaining milk stability, increasing its shelf life, and preventing the transmission of milk-borne diseases [2]. Pasteurized milk, as an intermediate product, is vulnerable to contamination.

Following a cross-contamination event, pasteurized milk may undergo quality degradation, eventually affecting the final processed product quality, and it may be at risk of harboring food-borne pathogens. Notably, *Listeria monocytogenes* comes at the forefront of pasteurized milk-transmitted pathogens [3]. Thus, one of the safety precautions adopted by the dairy plants is to impose a ceiling on the maximum duration of storage (>12 h).

Facing a rising consumer demand for fresh-tasting milk products with excellent nutritional attributes, the dairy industry has been compelled to ensure high-quality milk with little heat treatment, hence the supplementation of milk with additives. The consumers’ negative perception of chemical food preservatives has encouraged modern dairy industries to focus on natural additives, especially those of plant origin, as potential safer substitutes for synthetic compounds.

Ammoides (*Ammoides pusilla* L.), thyme (*Thymus vulgaris* L.), and rosemary (*Rosmarinus officinalis* L.) are widespread Mediterranean medicinal plants that have been well recognized since ancient times for having various medicinal and nutritional uses. These plants possess multiple health benefits and pharmacological properties, such as antioxidant [4,5], antimicrobial [6,7], and antitumor [8] properties.

Thyme and rosemary plants; their derivatives; and their pure compounds, such as carvacrol, thymol, and rosmarinic acid, have been used in the food industry to preserve and improve foods’ sensory and functional properties [9,10]. Ammoides plants, on the other hand, culinary herbs that have been proven to be non-toxic and safe [11], have not yet been tested for potential applications in food.

Regarding the above mentioned, this study aimed to assess the potential ability of medicinal plants (thyme, ammoides, and rosemary) aqueous extracts to enhance the pasteurized milk microbial stability during intermediate storage and attribute to its antioxidant functionality.

## 2. Results and Discussion

### 2.1. Extracts Phytochemicals Contents and Antioxidant Activities

Our investigation into the total phenolic, flavonoids and tannins contents (TPC, TFC, and CTC) of thyme, rosemary, and ammoides aqueous extracts revealed consistent differences (*p* < 0.05) among the plant extracts (Table 1). The highest and the lowest phenolic content were obtained in thyme and rosemary at 104.23 and 74.49 mg GAE/g of extract, respectively. The same pattern was observed with regard to TFC.

The thyme TPC value ranges from 7.3 to 158–256 mg GAE/g, as reported by the literature [12,13]. As for ammoides extracts, the recorded TPC value was distinctly lower than the value reported by Belkhodja1 et al. (2020) [11] in their study conducted on ammoides aqueous infusion. The different extracts have shown rich flavonoid content, representing more than 50% of the phenolic compounds. A significant correlation (r = 0.861, *p* < 0.05) between TPC and TFC was also noted. This outcome suggests that flavonoids are the main class of phenolic compounds in the different herbs. Regarding the extracts’ tannin content, rosemary and thyme exhibited the highest contents with respective values of 10.98 and 10.07 mgCE/g Ext.

Table 1 shows the antioxidant capacity assays’ results. Overall, all the extracts displayed an antioxidant potential, namely free ABTS/DPPH radical scavenging activities, reducing power (RP), and total antioxidant capacity (TAC). The different assay outcomes revealed that thyme exhibited the highest antioxidant potential, as is consistent with the phenolic quantification results. A significant strong correlation was recorded between the phenolic and flavonoid contents of the extracts and the different antioxidant activity assays (r absolute value ranges from 0.7 to 0.99).

Previous studies have reported a strong relationship between antioxidant activity and total phenolic content in vegetables, fruits, and medicinal plants [14]. Phenols’ structure, consisting of an aromatic ring bearing hydroxyl substitutes, explains their ability to act on oxidation through hydrogen atoms or electron donation, free radicals scavenging, or metal cations chelating [15]. Rosemary has shown the lowest DPPHand the lowest ABTS scavenging activities with respective IC_50_ values of 259.16 and 104.55 µg/mL. In contrast, the ammoides plant has shown both the lowest TAC and the lowest RP values of 30.12 and 154.79 mg AA/g Ext, respectively.

### 2.2. Extracts Phenolic Profile

The quantitative determination of the identified phenolic compounds in the aqueous extracts of thyme, rosemary, and ammoides is summarized in Table 2. Our characterization of the different extracts’ phenolic profiles revealed 24 phenolic compounds presented at different concentrations among samples.

It can be seen from Table 2 that the main common phenolic acids between the three extracts were ferulic, sinapic, caffeic, chlorogenic, and rosmarinic acids. The highest level of rosmarinic acid was recorded in ammoides extract (116 mg/100 g extract). Ferulic and rosmarinic acids were the main phenolic acids of rosemary, with respective concentrations of 97.53 and 74.64 mg/100 g extract. Ellagic acid, only identified in thyme extract, was its predominant phenolic acid.

Phenolic acid compounds have been proven to possess the potential to prevent several diseases associated with oxidative damage, such as cancer and heart disease [16]. Sinapic, the predominant phenolic acid identified in all samples, was proven to exhibit various biological properties, such as antioxidant and anti-inflammatory activities, and to be effective against other oxidative-related diseases [17].

Rosmarinic acid was reported to be the predominant phenolic compound in rosemary, thyme, and other *Laminacea* plants [18].

As for flavonoids, high levels of catechin hydrate, epicatechine-3-O-gallate, and isorhamnetin-3-O-glucoside were recorded in the different extracts. Rosemary exhibited an exceptionally high level of catechin hydrate (1222.94 mg/100 g extract). Rutin was the main flavonoid compound identified in thyme extract. Rutin, isorhamnetin-3-O-rutinoside, quercetin, and kaempferol were only identified in thyme extract. It should be noted that rosemary and thyme, both belonging to the *Lamiaceae* family, have shown similarities in their phenolic profiles.

Vallverdú-Queralt et al.’s (2014) [19] study on different culinary herbs, including thyme and rosemary, reported similar profiles to the ones obtained in our study, with several common main compounds, such as coumaric, rosmarinic, ferulic, caffeic, and syringic acids. However, the reported levels were not in line with our study results. Several factors related to the plant genetic diversity, environmental conditions, and growing stages may explain the deviation from the previously reported results [20].

### 2.3. Total Viable and Psychrotrophic Bacteria Count in Milk Supplemented with Plants Extracts

#### 2.3.1. Heat-Treated Supplemented Raw Milk

**Figure 1:**
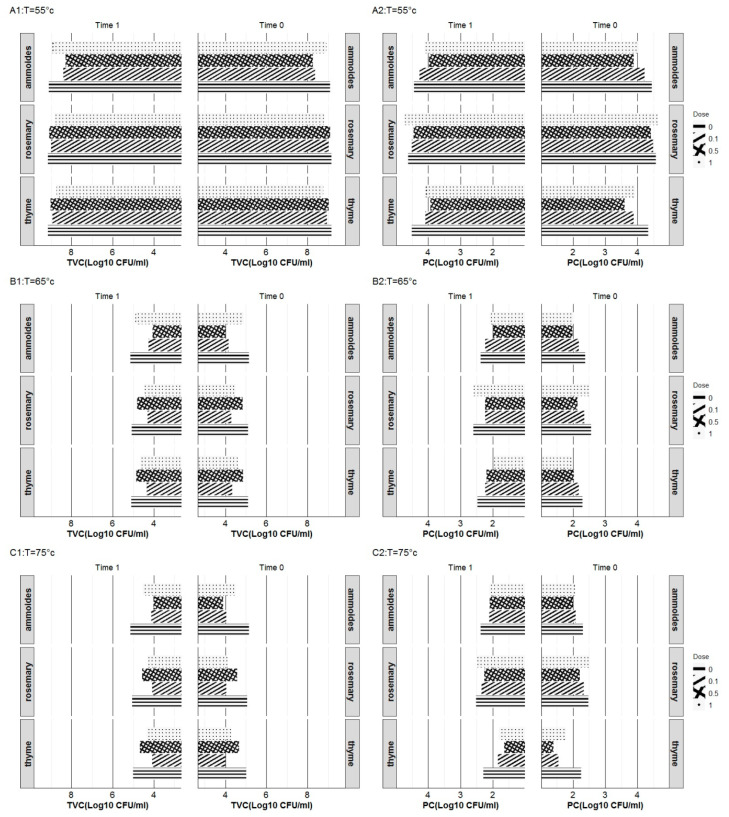
Total viable count and psychrotrophic bacteria count (TVC (1) and PC (2)) of thyme, rosemaryand ammoidesextractssupplemented milk sample, heat-treated at 55 °C (A1-2), 65 °C (B1-2), and 75 °C (C1-2). Aqueous extracts were added to milk at different levels (0, 0.1, 0.5, and 1 mg/mL).The bacterial countingwas carried out directly after heat treatment (Time 1) and after 12 h of refrigerated storage at 6 °C (Time 2).

Both the total viable count and psychrotrophic bacterial count (TVC and PC) of heat-treated and subsequently overnight-stored (at 6 °C) raw milk samples are shown in Figure 1.

The raw milk used in this study exhibited poor microbial quality. The milk primary TVC count was higher than 10.4 Log CFU/mL, which explains the atypically high TVC count, ranging from 8.22 to 9.07 Log CFU/mL of milk samples processed at 55 °C. The processing temperature significantly affects the milk bacterial count (*p* < 0.001) (Table 3).

The microbial counts (TVC and PC) considerably decreased at higher heating temperatures (65 and 75 °C).

The addition of herbal extracts to raw milk led to a reduction in bacterial viability. The supplemented samples exhibited lower bacterial counts than those shown by control samples for the different treatment groups.

Plant extracts’ antimicrobial activity is induced mainly by their phenolic components. However, their antimicrobial action mode and their primary targets are poorly understood. As complex natural mixtures, plant extracts’ potential antibacterial mechanism is thought to be a synergy between several mechanisms, such as cell membrane permeability alteration, enzyme inactivation, DNA synthesis inhibition, and protein denaturation [21].

The extract supplementation differently affected the psychrotrophic and the mesophilic bacterial population. Psychrotrophic bacteria were the most sensitive to ammoides extract. No prominent effect on the PC charge was observed following the supplementation of milk with rosemary extract. Compared to the control sample, rosemary supplemented samples have shown the lowest reduction levels (<0.3 Log CFU/mL). As for mesophilic bacteria, thyme extract has proven to be the most effective extract, reducing the TVC count by 0.11 to 0.85 Log CFU/mL. The different behavior of the two bacterial populations toward the used extracts may be explained by their different levels of sensitivity to the antimicrobial compounds contained in the extracts. Patricia et al. (2012) [22] have reported a significant difference in the antibiotic-resistance relevance of psychrotrophic and mesophilic bacterial populations in refrigerated raw milk.

Overall, the statistical analysis results have shown a significant effect of the interaction of the different factors (*p* < 0.001) (Table 3).

The reduction in the TVC of extracts supplemented milk was more pronounced as the processing temperature increased, regardless of the extract used. As for the PC, the temperature effect on the extract-supplemented samples depended on the extract added.

The observed behavior may be attributed to the interaction of the bioactive compound with milk components, fat, and protein. The temperature was proven to affect the protein–polyphenol interactions and, consequently, to play a key role in plant-extract functionality in a dairy food matrix [23]. According to Ozdal et al. (2013) [23], increasing the temperature could either favor or hinder the association between phenolic compounds and protein.

Regarding the supplementation levels, they differently affected the TVCs and PCs. Their effectiveness depended on the extract added and the processing temperature. The highest PC reduction levels in the different sample groups were at 0.5 mg/mL. As for the TVC, ammoides-extract milk supplementation was the most effective at 0.5 mg/mL, regardless of the temperatures applied. In comparison, thyme-extract milk supplementation was the most effective at 1 mg/mL at a low processing temperature (55 °C) and at 0.1 mg/mL at high processing temperatures (65 and 75 °C), as is also relevant to rosemary extract milk supplementation.

Following overnight storage, the bacterial counts (TVC and PC) of milk samples have shown, despite the slight variation, a similar pattern to the values recorded immediately after heat treatments (Figure 1). The interaction between the extract, time, and processing temperature was tested and found to be insignificant (*p* = 0.327).

#### 2.3.2. Cold-Stored Supplemented Pasteurized Milk

**Figure 2:**
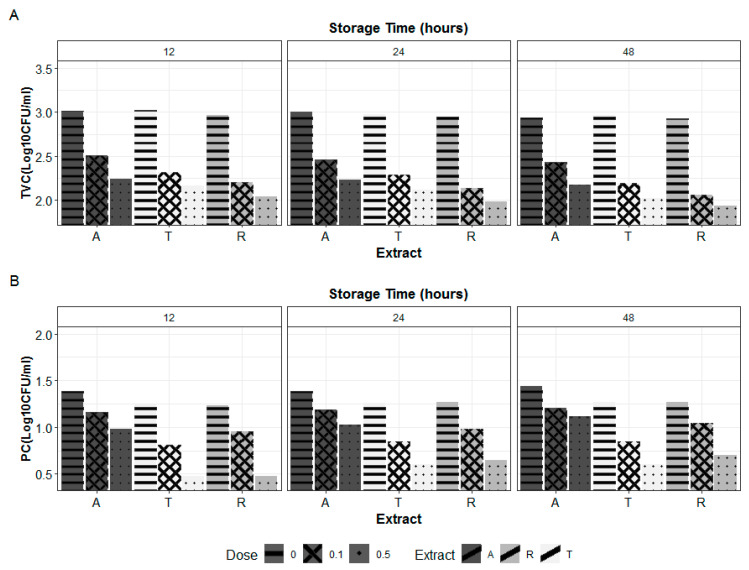
Total viable count (TVC, (**A**)) and psychrotrophic bacteria count (PC, (**B**)) of cold-stored (6 °C) pasteurized milk samples supplemented with thyme, rosemary, and ammoides aqueous extracts at different levels (0, 0.1, and 0.5 mg/mL). A, ammoides; R, rosemary; T, thyme.

Total viable and psychrotrophic bacterial counts (TVC and PC) of pasteurized milk samples stored at 6 °C are illustrated in Figure 2. The average PC and TVCinitial counts of control samples were 25 and 10^3^ CFU/mL, respectively.

The microbial load in pasteurized milk may be attributed to the growth of heat-resistant bacteria that are able to withstand the temperature of pasteurization [24].

The PC and TVC showed the same pattern at different sampling times. The storage time did not significantly affect the microbial charges; *p*-values, corresponding to time, extract, and dose interaction, were 0.094 and 0.154 for TVC and PC counts, respectively (Table 3). Cold storage was as effective for hurdling the microbial growth during a storage period of 48 h; the cooling stress on microorganisms results in the deactivation of the microbial growth, the reduction in the multiplication rate, and the enhancement of the lag phase [25].

Milk supplementation with the herbal extracts induced a significant reduction in the microbial charges. The reduction in TVC levels was higher than that recorded in PC levels for the different treatment groups. The PC has shown to be less affected by extracts supplementation. This observation may be explained by the fact that psychrotrophic bacteria are able to adapt to extremely low temperatures and are less affected by cooling stress [25].

It is worth mentioning that milk psychrotrophic bacteria constitute a significant microorganism group responsible for milk spoilage. By producing thermostable lipases and proteases, they are suspected to reduce the shelf life of both pasteurized and UHT milk [26]. As shown in Figure 2, the highest microbial numbers reduction was attained in samples supplemented with thyme for PC count with an average value of 0.56 Log cycle UFC/mL. As for the TVC count, rosemary-extract supplementation induced the highest reduction in TVC count by an average value of 0.89 Log cycle. Samples supplemented with ammoides extract exhibited the highest microbial charges (PC and TVC) among the fortified samples. Increasing the supplementation dose resulted in the improvement of the different extracts’ antimicrobial effects.

### 2.4. Anti-Listerial Activity in Milk

This study assessed the effect of the selected plant extract (thyme extract) effect on *L. monocytogenes* survival in pasteurized milk. Thyme extract was especially noted for this test since it has shown the greatest effect on the psychrotrophic bacteria count.

**Figure 3:**
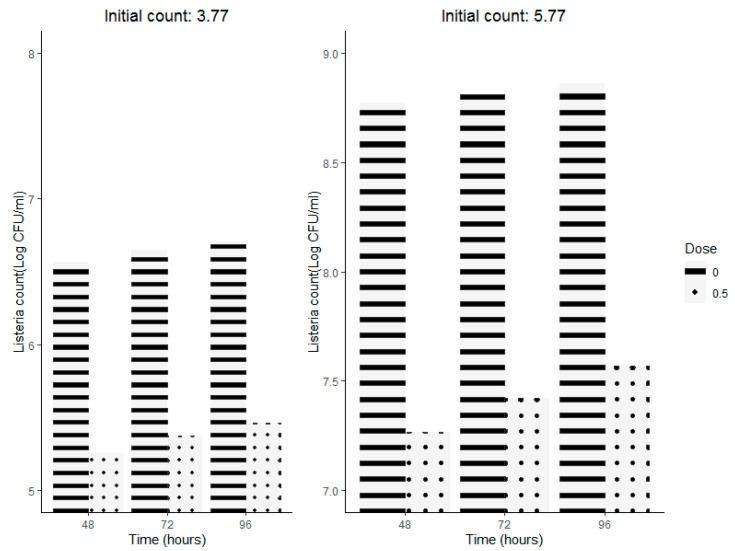
Growth of *Listeria monocytogenes* ATCC 19116in cold-stored (6 °C)milk inoculated at two different initial bacterial loads (3.77 and 5.77 Log CFU/mL) and supplemented with thyme aqueous extract (0.5 mg/mL).

The initial inoculated pathogen was 3.77 and 5.77 Log CFU/mL, and respective controls were counted at 6.56 and 8.7 Log CFU/mL, after a two-day storage period (Figure 3).

Thyme-extract treatment at a concentration of 0.5 mg/mL resulted in a significant anti-listerial activity, regardless of the initial microbial load. Colony count differences, as compared to control ones, ranged from 1.24 to 1.5 Log CFU/mL during a four-day storage period.

Several plant extracts have shown a significantly similar inhibitory effect on *L. monocytogenes* in milk, such as acid-hydrolyzed *Citrus unshiu* peel extract [27] and garlic sprouts juice [28]. The antimicrobial effectiveness of plant extracts is attributed in huge part to their phenolic compounds. The hydroxyl group enables the phenolic compounds to adhere to the bacterial cell membrane. The phenolic compounds may afterward induce the cell destruction by affecting the membrane permeability and integrity or by intervening with the intracellular enzyme functionality. The anti-listerial activity of phenolic compounds is mainly associated with the membrane-disruption mechanism [29].

### 2.5. Antioxidant Activity of Milk Serum

The DPPH scavenging activities of the control and fortified samples assessed after seven days of cold storage are summarized in Table 1. The supplemented milk exhibited a significantly higher radical scavenging capacity compared to the respective control. The antiradical activity of treated milk at 0.1 mg/mL decreased in the following order: thyme > rosemary > ammoides. Non-significant differences were recorded in fortified milk samples at 0.5 mg/mL. Thyme extract was the extract with the highest phenolic contents and the highest antioxidant activities.

Significant strong correlations were recorded between the milk serum radical-scavenging activity and the used extracts’ tannin content (CTC) and extract ferric-reducing power (RP) with respective correlation coefficients of 0.99 and 0.69. Thus, we can associate the enhancement of the antioxidant activity with the enrichment of the phenolic content of milk by adding the plant extracts, as they are in concordance with the higher radicals’ inhibition rate observed at higher supplementation concentrations.

Our results are in agreement with those reported by Reference [30]. The results of the abovementioned study have revealed an improvement in the antioxidant properties of milk supplemented with thyme essential oil in a free and encapsulated form. They explained these results by the ability of essential oil to preserve the integrity of milk proteins against bacteria-induced deterioration.

### 2.6. Sensory Evaluation

**Figure 4:**
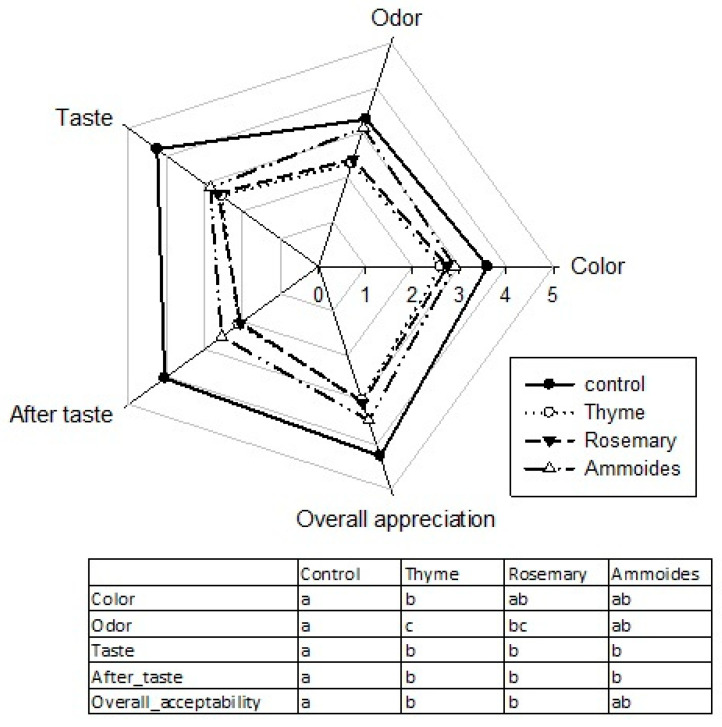
Radar chart for sensory evaluation of pasteurized milk samples. Samples: control, aqueous extracts supplemented pasteurized milk at 0.5 mg/mL (thyme, rosemary, and ammoides). The attached table represents the significance letters resulting from a Tukey’s post hoc test.

Sensory properties are the main quality attributes of foods affecting consumer behavior. Figure 4 shows a radar map of the sensory attributes’ appreciation scores for the different milk samples.

The appreciation scores were the highest for control samples regarding the different sensory attributes. A non-significant difference of taste and after-taste appreciation scores between the fortified milk samples was found. The extracts-supplemented milk samples taste and after-taste sensory characteristics were statistically equal among them and were the least appreciated compared to the control. However, their mean liking scores higher than 2, show that they are quite acceptable by consumers. Milk samples supplemented with ammoides extract exhibited the highest average liking score among the treated samples. Their scores were non significantly distinct from the control sample regarding the different sensory attributes, except for taste and after-taste. Thus, we can conclude that milk samples with the added ammoides extract were well appreciated by consumers.

## 3. Materials and Methods

### 3.1. Extracts Characterization

#### 3.1.1. Plant Materials and Aqueous Extracts Preparation

Ammoides blooming aerial part (*Ammoides pusilla* L.), thyme (*Thymus vulgaris* L.), and rosemary (*Rosmarinus officinalis* L.) leaves were purchased from a medicinal herbs market based in Tunis (Tunisia). Extract preparation was conducted according to Miljenko et al.’s (2016) method, but with some modification [31]. The dried and powdered plant materials (20 g) were stirred with 200 mL of distilled water at room temperature for 4 h and then brought to a standstill at 4 °C for 24 h. After filtration through a Whatman filter paper No.4, extracts were vacuum-evaporated to dryness.

#### 3.1.2. Extracts’ Phytochemicals Contents

The total phenolic content (TPC) of the aqueous extracts was determined by following the Folin–Ciocâlteu method [32]. The results were expressed as milligrams of gallic acid equivalents (GAE) per g of dry extract (mg GAE/g Ext).

The total flavonoid content (TFC) was assayed by using the Olatunji and Afolayan (2019) [33] method and expressed as milligrams of catechin equivalents (CEs) per g of dry extract (mg CE/g Ext).

The determination of the condensed tannins content (CTC) of extracts was carried out by the vanillin spectrophotometric method according to the protocol of Aydi et al. (2020) [34]. The results were expressed as milligrams of catechin equivalents (CEs) per g of dry extract (mg CE/g Ext).

#### 3.1.3. Antioxidant Activities Evaluation

Four different in vitro assays were conducted: scavenging effects on DPPH (2,2-diphenyl-1-picrylhydrazyl) and ABTS (2,2′-azinobis-(3-ethylbenzothiazoline-6-sulfonic acid) radicals, reducing power (following the ferricyanide Prussian blue assay), and total antioxidant capacity. The antioxidant activities were assessed following the experimental methodologies applied in the studies of Mokrani and Madani (2016) [35], Olatunji and Afolayan (2019) [36], Mahmud et al. (2017) [37], and Aydi et al. (2020) [38].

DPPH and ABTS scavenging assays assess the ability of the antioxidant compound to transfer labile H atoms and electrons to radicals, with DPPH being more selective toward hydrogen-donating compounds [39].

Radical-scavenging activities were calculated as a percentage of radical inhibition, using the formula: [(Ac − As)/Ac] × 100 (1), where As is the absorbance of the solution containing the sample, and Ac is the absorbance of the control. Results were given as (IC_50_), the concentration providing 50% of radical inhibition.

Reducing power (RP) and total antioxidant activity (TAC) were expressed as milligrams of ascorbic acid equivalents (AAE) per g of dry extract (mg AAE/g Ext).

#### 3.1.4. HPLC analysis of Phenolic Compounds

The aqueous extracts were analyzed by using an Agilent 1100 series HPLC system equipped with a diode array detector (Agilent Technologies, Waldbronn, Germany). The separation was performed on a Hypersil ODS C18 reversed-phase column (100 mm × 4.6 mm, 0.5 μm particle size) at a constant temperature of 23 °C.

Extracts were dissolved in methanol and chromatographed under gradient conditions, with a flow rate of 0.7 mL/min and an injection volume of 20µL. The mobile phase consisted of acetonitrile (mobile phase A) and 0.2% (*v*/*v*) water: formic acid (mobile phase B). The gradient starting with 65% of the B phase was kept constant for 6 min, and then the B phase concentration decreased according to the following order: 40% (6–9 min), 20% (9–14 min), 0% (14–25 min), and finally set back to its initial value65% (25–30 min). The chromatogram was acquired at 280 nm. The retention time of pure standards was used for the identification of the phenolic compounds, and the calibration curves were used for their quantification [40].

### 3.2. Fortified Milk Analysis

#### 3.2.1. Fortified Milk Samples Preparation

Raw and pasteurized milk (80 °C, 15 s) used to prepare the different samples were taken from bulk tanks at a local dairy plant (Tunisia).The raw milk’s composition was as follows, in %: fat (4.40),lactose (4.30),protein (3.50),SNF (8.15). A thoroughly mixed milk sample was transferred to sterile test tubes for up to 10 mL. Ammoides, rosemary, and thyme extracts’ stock solutions were prepared in distilled water and then filter-sterilized (filter with 0.2 µm poresize) before being added to milk at an amount of 1 mL to yield final concentrations of 0.1, 0.5, and 1 mg/mL. Control samples were added by1 mL of distilled sterile water.

#### 3.2.2. Fortified Milk Microbial Quality

The effect of plant extracts’ supplementation on the microbial stability of pasteurized milk was assessed through two experiments. These experiments were conducted with distinct goals:

For the first experiment, Experiment 1, extracts were added to raw milk prior to heat treatment and then stored under refrigeration for 12 h. Through this experiment, we aimed to answer whether extracts are effective if added prior to pasteurization and whether their effect depends on the applied heat treatment. As for the second experiment, Experiment 2, extracts were added to pasteurized milk and then stored under refrigeration for 48 h. This experiment aimed to answer whether extracts’ effect on the microbial quality of pasteurized milk would be maintained if we extended the storage period.

The detailed description of the experiments is as follows:

**Experiment** **1.** *Effect of milk fortification associated with heat treatment on milk microbial quality.*

Three lots, namely (A), (B), and (C), of samples prepared by using extract-enriched raw milk were subjected to heat treatment at 55, 65, and 75 °C, respectively. The samples of these lots were heated in a temperature-controlled water bath for 30 min and immediately cooled in an ice bath. Milk microbial quality tests were conducted after t = 0 and 12 h of incubation at 6 °C.

**Experiment** **2.** *Effect of cold storage on fortified pasteurized milk microbial quality.*

A fourth lot (D) of pasteurized milk samples, to which different extracts were added, were then stored at 6 °C. Milk microbial quality tests were conducted after t = 12, 24, and 48 h of incubation at 6 °C. Samples of the lot (D) were incorporated only at 0.1 and 0.5 mg/mL of each extract. Extract supplementation took place immediately after pasteurization.

The microbial quality of the samples lots (A, B, C, and D) was assessed by enumerating the total viable count (TVC) and the psychrotrophic count (PC) according to ISO methods (ISO 4833:2003; ISO 6730:2005). Microbial analysis was conducted in duplicate (two tubes for each treatment), and the average of the colony numbers were expressed as follows: (Log CFU/mL).

#### 3.2.3. Anti-Listerial Activity

One extract was chosen, based on the microbial-quality monitoring results, to assess its anti-listerial activity. The anti-listerial activity test was conducted as follows: Pasteurized milk was further sterilized at 120 °C for 15 min before use. Milk was then inoculated with two different initial populations of *L.monocytogenes* ATCC 19116 strain (3.77 and 5.77 Log CFU/mL). Either extract solution or distilled sterile water was then added to the 10 mL inoculated milk samples at a rate of 1 mL. The final concentration of extract in the treated sample was 0.5 mg/mL. The numbers of viable bacteria in treated samples and controls were enumerated after 48, 72, and 96 h of refrigerated storage at 6 °C. The colonies were counted on a Mueller Hinton plate incubated at 37 °C for 24 h.

#### 3.2.4. Antioxidant Activities of Fortified Milk Serum

Samples prepared using pasteurized milk, to which the different extracts were added at 0.1 and 0.5 mg/mL and stored at 6 °C for seven days, were used to assess the antioxidant property of fortified milk.

Milk samples were first acidified up to pH 4.6 to precipitate caseins. After centrifugation at 5000× *g* for 5 min at 4 °C, the middle layer was collected (defatted milk serum). The pH of milk serum was restored to its initial value (6.5), using NaOH solution (1 N). The antioxidant activity of milk serum samples using the DPPH method was determined as previously described. The results were expressed as radicals’ inhibition rate.

#### 3.2.5. Sensory Evaluation

The consumer acceptance of pasteurized milk fortified with the herbs’ aqueous extracts at 0.5 mg/mL and the control sample (non-supplemented sample) was assisted by sensory evaluation. Color, odor, taste, after taste, and overall acceptability were evaluated by a panel of 20 untrained random subjects of both genders (10 males and 10 females), using the five-point hedonic scale. The tested samples were prepared the same day and stored for a day (4 °C) before the panel sensory evaluation.

Samples were served at room temperature (26 °C), under normal light conditions, in transparent cups marked with three-digit randomized codes. Panelists were provided with water for rinsing between samples.

### 3.3. Statistical Analyses

Statistical analysis was carried out with the SPSS software (SPSS Inc. Chicago, IL, USA). Means of microbial counts (TVC and PC) were compared by using the General Lineal Model (GLM) repeated measures by means of one-way within-subject designs. We used the sampling time as within-subject factors, and extract, incorporation rate, and heat treatment temperature as between-subjects factors. Univariate ANOVA was carried out, using Tukey’s *t*-test to compare the values of the extract phytochemicals contents, the antioxidant analysis results, and the sensory mean scores. Differences were considered as significant at *p* < 0.05. Pearson’s correlation coefficient was also calculated. Plots were generated with R program v.3.6.3 (R Core Team, 2020), using the ggplot2 (Wickham, 2016) package.

## 4. Conclusions

In summary, our results indicate that the incorporation of thyme, rosemary, and ammoides aqueous extracts has positively affected the microbiological quality of pasteurized milk. Our study also highlighted the effect of associating heat treatment with milk extract supplementation on the efficacy of extracts as a bio-preservative. A significant interaction effect of the applied temperature, extract type, and extract concentration on the microbial charge was recorded.

Milk supplementation resulted in a considerable improvement of its antioxidant activity. Extracts owed their effect on milk quality to their rich content of phenolic compounds possessing interesting phytopharmaceutical activities, such as ferulic, rosmarinic, caffeic, and ellagic acid. A sensory evaluation test revealed good acceptability of ammoides-supplemented milk among consumers.

The overall results provide an exciting potential for the future application of medicinal plants’ aqueous extract, especially the tested plants (ammoides, thyme, and rosemary) as natural alternatives to artificial preservatives. Nevertheless, the complexity of the food matrix and the interaction of its component with the biological actives compounds of extracts should be considered in order to achieve an optimal exploitation of plant extract potential.

## Figures and Tables

**Table 1:** Mean phenolic compounds and antioxidant activities of aqueous extracts of thyme, rosemary, and ammoides and antioxidant activity of fortified pasteurized milk serum ^1^.

	Trait						
Extracts	TPC	TFC	CTC	ABTS_IC50_	DPPH_IC50_	TAC	RP
Thyme	104.23 ^a^_(±4.79)_	73.91 ^a^_(±4.34)_	10.98 ^a^_(±0.86)_	24.56 ^c^_(±2.30)_	30.44 ^c^_(±4.79)_	459.50 ^a^_(±27.57)_	519.02 ^a^_(±5.16)_
Rosemary	32.65 ^c^_(±0.46)_	14.56 ^b^_(±0.78)_	10.07 ^a^_(±0.05)_	104.55 ^a^_(±4.57)_	259.16 ^a^_(±4.79)_	155.42 ^b^_(±12.37)_	53.54 ^b^_(±2.93)_
Ammoides	74.49 ^b^_(±0.85)_	22.15 ^b^_(±5.60)_	3.25 ^b^_(±0.05)_	37.86 ^b^_(±4.79)_	122.41 ^b^_(±1.63)_	154.79 ^b^_(±4.60)_	30.12 ^c^_(±0.17)_
	**Fortified milk serum DPPH (%)**						
	**Control**	**Thyme**		**Rosemary**		**Ammoides**	
**[Extract] = 0.1 mg/mL**	10.69 ^d^_(±1.67)_	74.84 ^a^_(±0.33)_		68.68 ^b^_(±0.11)_		45.91 ^c^_(±0.00)_	
**[Extract] = 0.5 mg/mL**	10.69 ^b^_(±1.67)_	85 ^a^_(±1.08)_		82 ^a^_(±1.00)_		83 ^a^_(±2.00)_	

^1^ Total phenolic content, TPC (mg gallic acid equivalentsg^−1^ of extract (mgGAE/g Ext)); condensedtannincontent, CTC (mg catechin equivalentsg^−1^ of extract (mgCE/g Ext)); total flavonoidcontent, TFC (mg catechin equivalentsg^−1^ of extract (mgCE/g Ext)); DPPH_IC50_ (1.1diphenyl-2-picrylhydrazine) free-radical scavenging activity (IC_50_(µg/mL)). ABTS_IC50_ (2.2′-azinobis-(3-ethylbenzothiazoline-6-sulfonic acid)) free-radical scavenging activity (IC_50_ (µg/mL)); ferric reducing power, RP (mg ascorbic acidequivalentsg^−1^ of extract (mg AA/g Ext)); total antioxidant capacity, TAC (mg ascorbic acidequivalentsg^−1^ of extract (mg AA/g Ext)). Means followed by the same letters are non-significantly different (*p* < 0.05).

**Table 2:** Polyphenol profile of aqueous extracts of thyme, rosemary, and ammoides (mg/100 gExtract) ^1^.

	Thyme	Rosemary	Ammoides
**Simple phenols:**			
Resorcinol	24.75	17.08	ND
Catechol	12.93	9.31	ND
**Phenolic acid:**			
Gallic acid	1.59	2.01	ND
Chlorogenic acid	**20.23**	**32.14**	**38.30**
Caffeic acid	**15.75**	**48.78**	**80.23**
Syringic acid	7.35	5.03	8.83
p-Coumaric acid	10.03	24.42	41.08
Sinapic acid	**135.14**	**37.97**	**64.13**
Ferulic acid	**177.71**	**97.53**	7.40
Rosmarinic acid	**49.76**	**74.64**	**116.53**
Ellagic acid	**357.99**	ND	ND
Trans Cinnamic acid	1.12	ND	ND
**Flavonoids:**			
Catechin hydrate	**131.96**	**1222.94**	**32.73**
Epicatechine-3-O-gallate	**51.93**	**57.21**	**74.39**
Isorhamnetin-3-O-glucoside	**32.03**	**13.46**	**22.41**
Rutin	**138.64**	ND	ND
Isoquerci0in	ND	**19.81**	**26.40**
Kaempferol-3-O-rutinoside	ND	8.72	15.24
Isorhamnetin-3-O-rutinoside	**33.64**	ND	5.87
Quercetin	**13.71**	ND	ND
Luteolin	4.29	1.42	ND
Kaempferol	**13.94**	ND	ND
**Stilbene:**			
Resveratrol	ND	21.27	31.15
**Unknown**	1683.45	2747.82	1409.38
**Total**	2917.94	4441.72	1974.07

^1^ ND: not detected.

**Table 3:** Results of General Lineal Model (GLM) repeated measures by means of one-way within-subject designs (Mauchly sphericity test was used to compare the within-subject effects) ^1^.

Factor	Associative Effect of Extracts and Heat Treatments				Extracts Effect on Stored Pasteurized Milk			
	TVC (Log CFU/mL)		TPC (Log CFU/mL)		TVC (Log CFU/mL)		TPC (Log CFU/mL)	
	F	*p*-Value	F	*p*-Value	F	*p*-Value	F	*p*-Value
**Test of between-subject effects**								
Extract	1458.558	<0.001 ***	647.267	<0.001 ***	150.682	<0.001 ***	324.574	<0.001 ***
Dose	10,007.332	<0.001 ***	277.660	<0.001 ***	3107.662	<0.001 ***	891.344	<0.001 ***
Temperature	865,519.019	<0.001 ***	18,535.986	<0.001 ***				
Extract * Dose	2145.419	<0.001 ***	36.844	<0.001 ***	29.530	<0.001 ***	34.607	<0.001 ***
Temperature * Extract	302.142	<0.001 ***	46.107	<0.001 ***				
Temperature * Dose	712.021	<0.001 ***	4.540	0.002 **				
Temperature * Dose * Extract	89.396	<0.001 ***	10.335	<0.001 ***				
**Test within subjects effects**								
Time	126.724	<0.001 ***	120.975	<0.001 ***	88.220	<0.001 ***	54.386	<0.001 ***
Time * Extract	7.474	0.002 **	24.130	<0.001 ***	1.596	0.219	3.286	0.035 *
Time * Dose	9.139	<0.001 ***	3.606	0.022 *	3.040	0.044 *	11.947	<0.001 ***
Time * Temperature	16.345	<0.001 ***	3.728	0.034 *				
Time * Extract * Dose	5.264	0.001 **	3.544	0.007 **	2.079	0.094	1.732	0.158
Time * Temperature * Extract	1.201	0.327	1.477	0.230				
Time * Temperature * Dose	3.293	0.011 *	1.675	0.155				
Time * Temperature * Dose * Extract	2.849	0.007 **	1.493	0.172				

^1^ Significance differences * *p* < 0.05, ** *p* < 0.01 and *** *p* < 0.001.

## Data Availability

The data presented in this study are available on request from the corresponding author.
